# Effects of open-skill exercise on executive functions in children and adolescents: a systematic review and meta-analysis

**DOI:** 10.3389/fnhum.2024.1495371

**Published:** 2025-02-04

**Authors:** Shunding Hu, Peng Shi, Ziyun Zhang, Xiaosu Feng, Kai Zhang, Teng Jin

**Affiliations:** ^1^School of Physical Education, Liaoning Normal University, Dalian, China; ^2^School of Physical Education, Shanghai University of Sport, Shanghai, China; ^3^School of Life and Health, Huzhou College, Huzhou, China; ^4^School of Athletic Performance, Shanghai University of Sport, Shanghai, China; ^5^School of Physical Education, Shandong University of Aeronautics, Binzhou, China; ^6^Department of Graduate Studies, Shenyang Sport University, Shenyang, China; ^7^School of Physical Education, Shandong University of Technology, Zibo, China

**Keywords:** executive functions, open skills, physical exercise, brain science, children and adolescents

## Abstract

**Background:**

The research on the intervention of open-skill exercise on the executive functions of children and adolescents still requires quantitative synthesis, and there is inconsistency in the effects of intervention by strategic and interceptive skills, which are sub-divided from open-skill exercise. Therefore, this study systematically explores the aforementioned issues and examines the potential moderating factors in the effects of open-skill exercise intervention on executive functions.

**Methods:**

Computer searches of the CNKI, WOS, PubMed, ScienceDirect and SPORTDiscus databases were conducted. Two researchers independently screened the articles and extracted data, and used the bias risk assessment tool recommended by the Cochrane Collaboration Network and the Methodological Index for Non- Randomized Studies (MINORS) scale to assess included randomized controlled trials (RCTs) and quasi-experimental designs (QEDs). Statistical analyses were performed using Stata 16.0 software.

**Results:**

A total of 16 articles and 17 studies were included, comprising 11 RCTs and 6 QEDs. The participants were 1,298 children and adolescents aged 5 to 16. Open skill exercises have significant intervention effects (*p* < 0.01) on inhibitory control (*SMD* = −0.627, 95%*CI* = −0.890 to −0.363), working memory (*SMD* = −0.517, 95%*CI* = −0.768 to −0.266), and cognitive flexibility (*SMD* = −0.652, 95%*CI* = −1.085 to −0.219). The effects of strategic skill exercises are higher than those of interceptive skills, particularly in the dimension of inhibitory control (*SMD* = −0.707, 95%*CI* = −0.819 to −0.594, *p* < 0.05). In addition, moderate-intensity and higher-frequency exercises overall have a more positive effect on promoting executive functions (*p* < 0.05); interventions of 6 to 10 weeks are more effective for working memory (*p* < 0.05), while 30-min sessions are the most effective for working memory (*p* < 0.05), and sessions lasting 75 to 120 min are the most effective for cognitive flexibility (*p* < 0.05). Open-skill exercise has a more positive impact on inhibitory control in the 5–9 age group and on working memory in the 10–16 age group (*p* < 0.05); open-skill exercise, especially, has a more positive intervention effect on inhibitory control in the Eastern group (*p* < 0.05). Both Egger linear regression analyses and literature sensitivity analyses suggested that the Meta-analysis results were stable and reliable.

**Conclusion:**

Open-skill exercise has a positive intervention effect on executive functioning in children and adolescents, and strategic skill exercise interventions are more effective. In addition, the quantitative elements of exercise (intensity, frequency, and duration per session) and demographic factors (age and ethnicity) play a potential moderating role in this context. Based on this, it is recommended that children and adolescents choose more strategic open-skill exercises according to their actual situation and select appropriate quantitative exercise factors to maximize the enhancement of their executive functions.

**Systematic review registration:**

https://www.crd.york.ac.uk/prospero/#myprospero, CRD42025636714.

## 1 Introduction

Executive functions are goal-oriented, complex task-solving cognitive processes (Friedman and Robbins, 2022; Funahashi, 2001), and are a multidimensional cognitive structure composed of inhibitory control, working memory, and cognitive flexibility (Baddeley, 1996; Pennington and Ozonoff, 1996). Executive functions play an important role in academic performance and innovative thinking (Guo et al., 2019; Zhang, 2021), the development and maintenance of healthy behaviors (Allan et al., 2016; Yang et al., 2020), as well as mental health and social adaptation (Liu et al., 2021; Xie et al., 2020). The childhood and adolescence period is the golden age for the development of executive functions. Research on strategies to promote executive functions during this stage is of great importance for future real-life decision-making, everyday reasoning, and the establishment of good interpersonal relationships (Shi et al., 2023c).

Physical exercise is a natural means of promoting the development of executive functions in children and adolescents (Verburgh et al., 2014; Xue et al., 2019). Currently, research on the relationship between physical exercise and executive functions mainly focuses on the exploration of dose-effect relationships. A series of studies (De Greeff et al., 2018; Rathore and Lom, 2017; Song et al., 2022; Xie, 2020) have found that long-term regular, moderate frequency and duration, and moderate-intensity exercise have the best intervention effects on the executive functions of children and adolescents. In addition, the type of exercise is an important qualitative element of exercise intervention and is also an important element that researchers (Diamond and Ling, 2016; Tomporowski et al., 2015) call for further exploration in studies. Researchers have conducted meta-analyses on the effects of aerobic (Song et al., 2022) and high-intensity interval training (Hu et al., 2021) interventions on the executive functions of children and adolescents, and both have found positive intervention effects. Furthermore, current meta-analyses on resistance training interventions for executive functions are mostly derived from the middle-aged and elderly population (Coelho-Junior et al., 2022; Landrigan et al., 2020), possibly due to misconceptions about the safety of resistance training for children and adolescents and its adverse effects on bone development.

Motor skills are the carriers of physical exercise, representing the combination of psychological processes and skill operation processes, and there is a shared brain mechanism for skill learning and cognitive tasks (Meijer et al., 2022; Ji et al., 2010). As research progresses, scholars (Gu et al., 2019; Shi and Feng, 2022; Shi et al., 2022) have gradually focused on the relationship between types of motor skills and the executive functions of children and adolescents. Based on the unpredictability of the environmental context, motor skills can be divided into open and closed skills. The former refers to the skills of performing movement tasks in an unpredictable environment, requiring individuals to respond and adjust their movements according to changes in the environment; the latter refers to the skills of performing movement tasks in a stable, predictable environment, where individuals can pre-plan their movement procedures (Zhang, 2012).

The aforementioned studies (Gu et al., 2019; Shi and Feng, 2022; Shi et al., 2022) all found that the effect of open-skill exercise intervention on the executive functions of children and adolescents is better than that of closed skills, mainly due to the rich environmental stimuli of open skills, which can generate more cognitive load on the brain (Shi et al., 2023c). In addition, there is a certain degree of similarity in cognitive demands between open-skill exercise and exercise that involves motor and cognitive dual tasks. The former requires participants to adapt to a constantly changing environment, necessitating greater cognitive load and promoting targeted practice of certain cognitive functions; the latter requires participants to perform cognitive tasks while engaging in physical activities. Both can enhance neuroplasticity, stimulate the formation of new neural connections, improve the integration of sensory and motor processes, and promote cognitive development (Deodato et al., 2024; Shi and Feng, 2022; Wollesen et al., 2022). This provides further evidence to support the positive benefits of open-skill exercise. However, some studies (Becker et al., 2018; Chueh et al., 2017) have reported that there is no difference in the effects of open and closed-skill exercise interventions.

The aforementioned controversy may be due to the differences in various types of open-skill exercises. According to Voss et al. (2010), open skills are further divided into strategic skills and interceptive skills. Strategic skills require individuals to process a large amount of information from the identities, positions, speeds, and trajectories of offensive and defensive players simultaneously, such as in soccer, basketball, hockey, and ice hockey. Interceptive skills require participants to coordinate dynamically with objects in the environment using their body, body parts, or handheld equipment, such as in table tennis, tennis, fencing, and boxing. Krenn et al. (2018) compared the executive function performance of athletes with interceptive, strategic, and closed skills. The results showed that compared to athletes with closed skills, those with strategic skills exhibited unique cognitive advantages in all three sub-functions of inhibitory control, working memory, and cognitive flexibility. In contrast, athletes with interceptive skills only performed well in inhibitory control. However, Voss et al. (2010) showed that athletes with interceptive skills had better cognitive performance than those with strategic skills. Therefore, the relationship between strategic skills and interceptive skills and executive functions is still inconsistent. Research on strategic and interceptive skills is mainly focused on athlete populations, while research on children and adolescents is relatively scarce. Moreover, it is necessary to further explore which type of open-skill exercise intervention is more effective under the premise of maximizing intervention strategies, thus necessitating subsequent studies to further explore based on this classification system.

Based on this, this study systematically searches for literature related to the intervention of open-skill exercises on the executive functions of children and adolescents, and on this basis, uses the method of combining effect sizes to test the effectiveness of the intervention, as well as to further explore the differences in the intervention effects of strategic and interceptive-skill exercises. In addition, this study also further explores the moderating effects of quantitative intervention elements and demographic variables in the intervention. Through this study, it is hoped to provide evidence for open-skill exercise interventions to improve the executive functions of children and adolescents, filling the research gap in strategic and interceptive skill exercises among children and adolescents, and providing a basis for formulating subsequent exercise intervention strategies for the executive functions of children and adolescents.

## 2 Literature review and research hypotheses

### 2.1 Effects of open skill-exercise on the executive functions of children and adolescents

Open-skill exercises contain a rich array of environmental stimuli and interpersonal interaction elements, as well as a variety of problem-solving target tasks, which present children and adolescents with more cognitive challenges and thus offer superior benefits for the promotion of executive functions (Shi et al., 2023c). Although some studies (Becker et al., 2018; Chueh et al., 2017; Jäger et al., 2015) have not highlighted the positive benefits of open-skill exercises in promoting the executive functions of children and adolescents, more studies results have confirmed the more active promoting benefits of open-skill exercises. A series of cross-sectional studies (De Waelle et al., 2021; Holfelder et al., 2020; Möhring et al., 2022) show that children and adolescents participating in open-skill exercises have more cognitive response advantages in executive function task performance, can more readily perceive the trend of cognitive conflicts, and consciously adjust cognitive control strategies to allocate more attentional resources to resolve conflicts (Larson et al., 2014). A series of experimental studies also found that open-skill exercises, which constitute cognitive challenges, have positive intervention effects on the attention inhibition (Gallotta et al., 2015), verbal working memory (O’Brien et al., 2021), spatial working memory (Ottoboni et al., 2021), and executive function (Bai et al., 2022; Crova et al., 2014) task performance of children and adolescents, and the benefits produced by open skills are superior to those of closed skills.

In addition, previous meta-analyses have also confirmed the promoting benefits of open-skill exercises on executive functions. For example, Shi et al. (2022, 2023d) showed that open skills significantly promote the executive functions of both typical and atypical children and adolescents. Zhang et al. (2022) used a web- based meta-analysis to assess the intervention effects of 11 types of motor skills on the working memory of school-age children, and overall presented a pattern of “open skills > closed skills.” Therefore, based on the above research findings, this study proposes *Research hypothesis 1*: open-skill exercises can significantly improve the executive function of children and adolescents.

### 2.2 Differences in intervention effects of strategic and interceptive-skill exercises

Voss et al. (2010) further categorized open skills into strategic and interceptive skills in their research on the cognitive advantages of elite athletes and found that athletes with interceptive skills had superior cognitive performance. However, more studies (Krenn et al., 2018; Yu et al., 2019) support that athletes with strategic skills have more active executive function task performance. For example, Yu et al. (2019) used near-infrared brain imaging technology to compare the behavioral and brain activation differences in the attentional executive control network functions of basketball (strategic skill), table tennis (interceptive skill), and track and field (closed skill) athletes. They found that basketball and table tennis athletes showed higher executive control advantages, accompanied by significant activation in the right prefrontal cortex and inferior frontal gyrus, but basketball athletes were accompanied by activation in more brain areas such as the frontal eye field.

In addition, the results of Voss et al. (2010) seem to be inconsistent with research on children and adolescents. Song et al. (2022) integrated randomized controlled trials of strategic game-dominated physical exercise on the executive functions of children aged 4 to 12 through meta-analysis and found that strategic game exercises have positive intervention effects on inhibitory control, working memory, and cognitive flexibility. However, some studies show that the sports environment of interceptive skills and strategic skills is different, and there are specific cognitive requirements, so there is a selective promotion effect on cognitive performance. For example, Wu et al. (2007) compared the intervention effects of football (strategic skill) and table tennis (interceptive skill) on the attention quality of primary school students and found that football exercise was better than table tennis in the intervention effects of attention maintenance and attention transfer; while table tennis exercise was better than football in the intervention effects of attention stability, attention breadth, and attention concentration. Ji (2014) compared the relationship between four types of open skills and the executive function task performance of primary school students and found that the basketball and table tennis groups performed best in the inhibitory control task, while the badminton and taekwondo groups performed best in the cognitive flexibility task.

In addition, Shi et al. (2022) found that the promotion effect of strategic skills on the executive functions of children and adolescents is better than that of interceptive skills. However, this study only compared the proportion of studies with promotional benefits in different types of skill exercises through frequency analysis, and it is not possible to determine the effect size of the intervention, which greatly reduces the accuracy of the research results. Based on previous research on children and adolescents, this study proposes *Research hypothesis 2*: There is a difference in the effects of strategic and interceptive skill exercises on the executive functions of children and adolescents, and the effect of strategic skill exercise intervention is better than that of interceptive skills.

### 2.3 Moderating role of quantitative elements in the effectiveness of open-skill exercise interventions

The quantitative elements of exercise intervention are the focus of dose–response relationship studies and an important factor for researchers to consider when formulating optimal intervention plans. These primarily include the intensity, duration, frequency, and duration of each exercise session.

Intervention intensity refers to the degree of physiological stimulation of the body activity in the exercise plan, which is mainly graded based on maximum heart rate or subjective feeling, and is usually divided into low intensity (<55% HRmax), moderate intensity (55–75% HRmax), and high intensity (>75% HRmax). Dong (2018) and Xie (2020) compared the intervention effects of moderate and high-intensity exercise on the working memory of children and adolescents, and found that moderate-intensity exercise has a positive intervention effect on both children and adolescents, while the intervention effect of high-intensity exercise is not significant, which may be related to the excessive fatigue induced by high-intensity exercise (Zhang, 2008). Shi et al. (2022), based on real exercise situations, also presented similar results, showing that both acute and long-term interventions of moderate exercise help to improve the executive functions of children and adolescents. In addition, research on the executive functions of atypical children and adolescents also shows similar results. For example, Yang (2021) found in a meta-analysis of exercise interventions for children with attention deficit hyperactivity disorder that moderate-intensity exercise has a positive intervention effect on inhibitory control and cognitive flexibility tasks. However, a recent meta-analysis (Feng et al., 2023) showed that both moderate and high-intensity open-skill exercises have a positive promotional benefit for the executive functions of typical children. But there is relatively less literature on high-intensity exercise, and the accuracy of its results needs further verification. Based on this, this study proposes *Research Hypothesis 3*: Intervention intensity has a moderating effect on the intervention effect of open-skill exercise on the executive functions of children and adolescents, and the intervention effect of moderate intensity is the best.

The intervention period is the total time from the start to the end of the exercise plan, usually measured in weeks. The human body gradually forms a response pattern adapted to the stimulus, suitable for its own survival, after long-term exposure to a specific stimulus (Wang and Su, 2016). Therefore, most current studies on the promotion of executive functions by exercise intervention are conducted with long-term interventions. Li et al. (2020) and Song et al. (2022) compared the promoting effects of different exercise intervention periods on the executive functions of children aged 3–7 and 4–12, respectively. The former showed that interventions below and above 10 weeks are both beneficial to the development of executive functions; while the latter showed that overall, interventions above 10 weeks have a better promoting effect. Shi et al. (2022) compared the promoting effects of real environment exercises below 8 weeks, 9–16 weeks, and above 17 weeks on the executive functions of children and adolescents through systematic review and found that interventions above 17 weeks have a more obvious effect. However, the study has not yet conducted a higher level of evidence combined effect test, so its accuracy is insufficient. Based on this, this study proposes *Research hypothesis 4*: The intervention period has a moderating effect on the intervention effect of open-skill exercise on the executive functions of children and adolescents, and the effect of longer period exercise is better.

The intervention frequency is the number of times the exercise plan is accepted within a unit of time, usually referring to the number of times of exercise per week. Li et al. (2020) showed that exercises below 3 times/week and above 3 times/week both have a positive intervention effect on the executive function task performance of children. However, Song et al. (2023) showed in their research on children and adolescents with attention deficit hyperactivity disorder that there is a selective promoting effect of exercise frequency on each dimension of executive function, that is, exercise at 2 times/week has a large effect size on the intervention effect of inhibitory control; exercise above 3 times/week has a medium to high effect size on the intervention effect of working memory. In addition, more studies support that the effect of exercise above 3 times/week is more positive, for example, Shi et al. (2022) and Song et al. (2022) showed that exercise above 3 times/week has a more positive promoting effect on the executive functions of children and adolescents. Based on this, this study proposes *Research hypothesis 5*: The intervention frequency has a moderating effect on the intervention effect of open-skill exercise on the executive functions of children and adolescents, and exercise above 3 times/week has a more positive promoting effect.

There is no unified standard for the classification of each exercise time in related studies, so there is no specific evidence of exercise time. Chen et al. (2015) found that 30 min of moderate-intensity basketball exercise has a better intervention effect on the executive functions of primary school students than 8 min and 15 min. Li et al. (2022) found that 40 min of moderate-intensity “basketball + rope skipping” intervention has a better promoting effect on executive functions than 20 min. In addition, related systematic reviews also show inconsistent results. Li et al. (2020) showed that exercises above and below 35 min both have a positive intervention effect on the executive functions of preschool children; while Song et al. (2022) showed that exercises lasting above 35 min have a higher effect size. Chang et al. (2012) showed that exercises lasting above 20 min have a more positive promoting effect on the cognitive task performance of adolescents; Yang (2021) found in his research on children with attention deficit hyperactivity disorder that exercises lasting 40–60 min overall have a higher effect size. In addition, Shi et al. (2022) also found that there is an inverted U-shaped relationship between each exercise time and executive function task performance, that is, exercises between 30–50 min have a better intervention effect on the executive functions of children and adolescents than those below 30 min and above 50 min. Based on this, this study proposes *Research hypothesis 6*: Each exercise time has a moderating effect on the intervention effect of open-skill exercise on the executive functions of children and adolescents, and moderate exercise time has a more positive promoting effect.

### 2.4 Moderating role of demographic variables in the intervention effects of open-skill exercises

Age, gender, and ethnicity are important demographic factors that affect the executive functions of children and adolescents (Peng et al., 2023). Firstly, executive functions have been regulating human activities since infancy, and some researchers (Anderson et al., 2018) have found that the fastest development stages of human executive functions are between 0 to 2 years, 7 to 9 years, and 16 to 19 years. Zhao et al. (2015) have shown that younger children have a more significant advantage in cognitive improvements caused by exercise. In addition, a meta-analysis (Wang et al., 2019) further confirmed this result, indicating that exercise has a positive effect on children, adolescents, and the elderly, but the effect size in the child group is much higher than in other groups. Secondly, girls generally exhibit higher executive functions and usually take longer than boys (Zhao et al., 2015). Moreover, boys are more inclined to participate in open-skill exercises with intense physical confrontation and rapid transitions of offense and defense, which is more conducive to the development of their executive functions. However, this study has not yet found comparative studies on the effects of exercise intervention on the executive functions of different gender groups. Lastly, Rea-Sandin et al. (2021) shows that the absolute difference in executive functions between white people and ethnic minorities is much larger than the absolute differences between ethnic minorities and within ethnic minorities, suggesting that ethnicity is a factor affecting executive functions. But this study has also not yet found comparative studies on the effects of exercise intervention on the executive functions of different ethnic groups.

Based on the above, this study proposes *Research hypothesis 7*: Age has a moderating effect on the intervention effect of open-skill exercises on the executive functions of children and adolescents, and exercise has a higher promoting effect on younger children than on older children and adolescents; and *Research hypothesis H8*: Gender has a moderating effect on the intervention effect of open-skill exercises on the executive functions of children and adolescents, and exercise has a more positive promoting effect on boys. In addition, for research on ethnicity, this study only conducts exploratory research and does not propose a research hypothesis.

## 3 Methods

This study was conducted in compliance with Preferred Reporting Items for Systematic Reviews and Meta-Analyses (PRISMA) 2020 and was registered at International Prospective Register of Systematic Reviews (PROSPERO), under number CRD42025636714.

### 3.1 Search strategies

A single researcher conducted a literature search using Chinese and English search terms. Search terms included: (1) motor skill, sports skill, sports items, exercise, physical activity, fitness, exercise; (2) executive function, working memory, inhibition control, cognitive flexibility, self-control, self-regulation; (3) children, child, adolescents, pupils, teenagers, young, students. Boolean logic operators AND were used to connect the three sets of search terms, and OR was used to connect similar search terms. Searches were conducted in the CNKI, Web of Science (WOS), PubMed, ScienceDirect, and SPORTDiscus databases, with the search period extending from the establishment of the database to December 2023.

### 3.2 Inclusion and exclusion criteria

Inclusion and exclusion criteria were designed according to the PICOS principle (Costantino et al., 2015). Inclusion criteria: (1) Participants were typically developing child adolescents; (2) Intervention measures are long-term interventions based on open skills; (3) Control measures include traditional physical education courses, basic academic courses, free activities, etc.; (4) Outcome variables include inhibition control, working memory, cognitive flexibility; (5) Study design includes randomized controlled trials (RCTs) and quasi-experimental designs (QEDs). Exclusion criteria: (1) Atypically developing children and adolescents with cognitive or intellectual disabilities, physical disabilities, etc.; (2) Unreported or uncertain types of motor skills; (3) Combination of open and closed skill interventions; (4) Screen-based motion games, such as Xbox, Kinect, and Nintendo, etc.; (5) Combined interventions of sports activities and cognitive therapy; (6) Cross-sectional, case–control, historical data, and other descriptive studies; (7) Reviews, abstracts, letters, comments, etc.; (8) Literature that cannot obtain original data (means and standard deviations); (9) Repeated publications for the same research subjects, only higher-quality literature is included. Two researchers independently conducted literature screening, and two other researchers conducted a second assessment of the screened literature. If there is controversial literature, the group discusses and decides together.

### 3.3 Data coding

This study is coded according to the following four parts: (1) Basic information of the literature, including the author, publication date, and research design; (2) Characteristics of the participants, including sample size, age, female proportion, and race; (3) Intervention and control measures, including intervention measures (intervention carrier, intervention intensity, intervention period, intervention frequency, and duration per session) and control measures; (4) Outcome variables, including measurement tools and main findings. In addition, the study classifies the intervention carrier into strategic skills and interceptive skills based on the classification standards of open skills. Included studies use reaction time, accuracy, and scores as three types of evaluation indicators to reflect the performance of executive function tasks. The faster the reaction time, the higher the accuracy and score, the better the executive function is indicated to be. To ensure the consistency of the direction of the index evaluation, the accuracy and score are extracted and coded in the opposite direction according to the study by Feng et al. (2023). The effect size is coded as an independent sample, that is, one independent sample codes one effect size. If there are multiple independent samples, they are coded separately (Cao et al., 2022).

Subgroup analysis helps to further clarify the effects of open skill exercise interventions on children and adolescents. Coding of moderating variables: (1) Open skill types are divided into strategic and interceptive skills; (2) Intervention intensity is divided into low, medium, and high; (3) Intervention period is divided into 6–10 weeks and 16–36 weeks; (4) Intervention frequency is divided into 1–2 times/week and 3–5 times/week; (5) Intervention time is divided into 30 min/session, 30-60 min/session, and 75-120 min/session; (6) Age is divided into 5–9 years old and 10–16 years old; (7) Race is divided into Eastern (including China and South Korea) and Western (including Italy, the United States, Switzerland, and Spain). Two researchers independently extract literature data, and two other researchers conduct a second assessment of the extracted content. If there are controversial issues, the group discusses and decides together.

### 3.4 Quality assessment

This study uses the bias risk assessment tool recommended by the Cochrane Collaboration Network (Cumpston et al., 2019) to assess the quality of RCTs. The tool assesses from six aspects: randomization methods, blinding, allocation concealment, completeness of outcome data, selective reporting of study results, and other biases. Additionally, the Methodological Index for Non-Randomized Studies (MINORS) scale (Slim et al., 2003) is used to assess the quality of QEDs. This tool includes 12 items, with 9 to 12 items used for additional criteria in evaluating studies with a control group, each item is worth 2 points, with a total score of 24 points. A score of 0 indicates not reported; 1 indicates reported but insufficient information; 2 indicates reported and provides adequate information. Two researchers independently make judgments based on the evaluation tools, and if there are serious disagreements on items, they will discuss with a third researcher.

### 3.5 Statistical analysis

Data processing and statistical analysis are conducted using Stata16.0. Firstly, a meta-analysis is used to explore the effects of open skill exercise interventions on the executive functions of children and adolescents. The meta-analysis uses the standardized mean difference (*SMD*) to represent the effect size and the 95% *CI* to represent the estimated interval of the overall parameters constructed by the sample statistics. The *Q* test and *I^2^* statistic are used to test the heterogeneity between studies. If *I^2^* < 50%, *p* > 0.1, it is considered that there is little heterogeneity between studies, and a fixed effect model is selected for analysis; if *I^2^* ≥ 50%, *p* ≤ 0.1, it is considered that there is significant heterogeneity between studies, and a random effect model is selected for analysis (Sun and Shi, 2021). Secondly, subgroup analysis and meta-regression analysis are used to explore the moderating effects of exercise intervention elements and demographic variables. Finally, the Egger linear regression model is used for literature publication bias test, and the one-by-one exclusion method is used for literature sensitivity analysis. The heterogeneity test level is set to *α* = 0.1, and the other test levels are set to *α* = 0.05.

## 4 Results

### 4.1 Screening results

**Figure 1:**
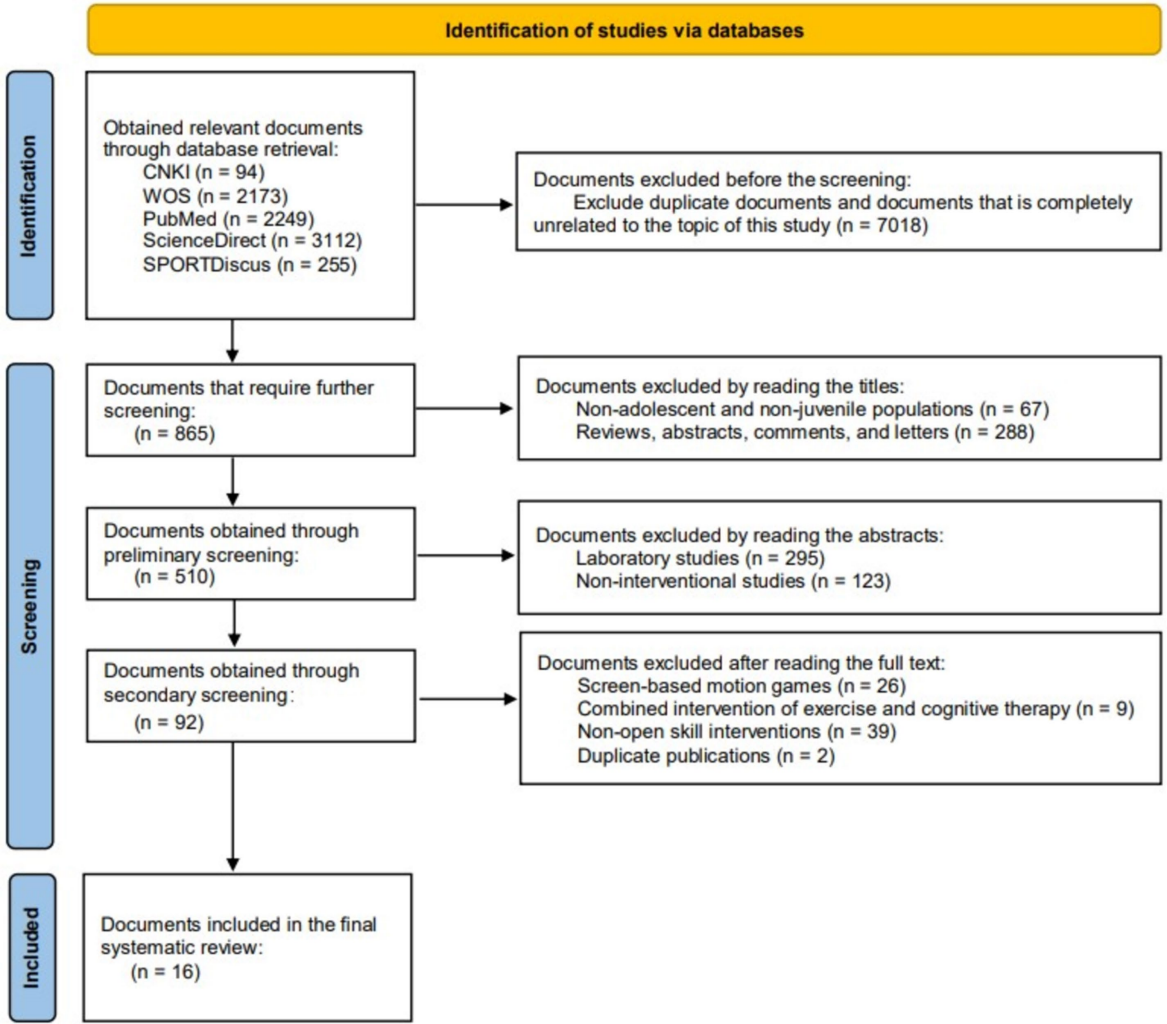
Flowchart of the screening process.

This study retrieved a total of 7,883 articles through the search. After deduplication using EndNote X9 software and excluding articles that were completely unrelated to the topic of this study (for example, those that did not include the search keywords in the title at all), 865 articles were obtained. In addition, based on the inclusion and exclusion criteria, several rounds of screening were conducted, and ultimately 16 articles were included. The search process is shown in Figure 1.

### 4.2 Basic characteristics of the included studies

#### 4.2.1 Bibliographic information and study design

Chang et al. (2013) explored the effects of low and moderate intensity exercise interventions. To estimate the effect size, this study extracted data from different intensity interventions in that literature, meaning the literature includes two sub- studies, thus a total of 17 studies were included. The publication dates of the included studies ranged from 2013 to 2022, including 11 randomized controlled trials (RCTs) and 6 quasi-experimental designs (QEDs).

#### 4.2.2 Characteristics of the participants

Included studies comprised a total of 1,298 participants, with the smallest sample size being 13 (Chang et al., 2013) and the largest being 460 (Pesce et al., 2016), averaging a sample size of 76. Participant ages ranged from 5 to 16 years old, with 9 studies involving participants aged 5 to 9 years (Chang et al., 2013; Crova et al., 2014; Jiang and Zeng, 2015; Alesi et al., 2016; Pesce et al., 2016; Chen et al., 2017; Lai et al., 2020; Ma et al., 2022), and 8 studies involving participants aged 10 to 16 years (Lakes et al., 2013; Schmidt et al., 2015; Martín-Martínez et al., 2015; Cho et al., 2017; Dai, 2020; Pan et al., 2016; Yin et al., 2017; Chou et al., 2020). The proportion of female participants in the included studies varied from 0.0 to 62.3%, with 5 studies (Jiang and Zeng, 2015; Dai, 2020; Ma et al., 2022; Pan et al., 2016; Yin et al., 2017) not reporting the proportion of female participants. Participant nationalities included China (Chang et al., 2013; Jiang and Zeng, 2015; Chen et al., 2017; Dai, 2020; Lai et al., 2020; Ma et al., 2022; Pan et al., 2016; Yin et al., 2017; Chou et al., 2020), Italy (Crova et al., 2014; Alesi et al., 2016; Pesce et al., 2016), the United States (Lakes et al., 2013), Switzerland (Schmidt et al., 2015), Spain (Martín-Martínez et al., 2015), and South Korea (Cho et al., 2017). There were 11 studies (Chang et al., 2013; Jiang and Zeng, 2015; Chen et al., 2017; Dai, 2020; Lai et al., 2020; Ma et al., 2022; Pan et al., 2016; Yin et al., 2017; Chou et al., 2020; Cho et al., 2017) involving Eastern populations and 6 studies (Crova et al., 2014; Alesi et al., 2016; Pesce et al., 2016; Lakes et al., 2013; Schmidt et al., 2015; Martín-Martínez et al., 2015) involving Western populations.

#### 4.2.3 Intervention and control measures

This study mainly adopts interventions through single sports such as soccer (Chang et al., 2013; Jiang and Zeng, 2015; Alesi et al., 2016; Chen et al., 2017; Dai, 2020; Ma et al., 2022), basketball (Pan et al., 2016; Yin et al., 2017), tennis (Crova et al., 2014; Lai et al., 2020), and taekwondo (Lakes et al., 2013; Cho et al., 2017). There are also studies (Schmidt et al., 2015; Martín-Martínez et al., 2015; Pesce et al., 2016; Chou et al., 2020) that use a variety of game-like sports to develop dynamic coordination and cognitive functions. According to the classification of open skills, there are 13 studies (Chang et al., 2013; Jiang and Zeng, 2015; Alesi et al., 2016; Chen et al., 2017; Dai, 2020; Ma et al., 2022; Pan et al., 2016; Yin et al., 2017; Schmidt et al., 2015; Martín-Martínez et al., 2015; Pesce et al., 2016; Chou et al., 2020) that use strategic skills for intervention; there are 4 studies (Crova et al., 2014; Lai et al., 2020; Lakes et al., 2013; Cho et al., 2017) that use interceptive skills for intervention.

In the included studies, 14 studies (82.35%) reported the intensity of the exercise intervention, among which one study (Chang et al., 2013) used low-intensity intervention; 12 studies (Chang et al., 2013; Crova et al., 2014; Jiang and Zeng, 2015; Martín-Martínez et al., 2015; Pesce et al., 2016; Chen et al., 2017; Cho et al., 2017; Dai, 2020; Lai et al., 2020; Pan et al., 2016; Yin et al., 2017; Chou et al., 2020) used moderate-intensity intervention; one study (Schmidt et al., 2015) used high-intensity intervention. The intervention period ranged from 6 weeks (Schmidt et al., 2015) to 36 weeks (Lakes et al., 2013), with 9 studies (Chang et al., 2013; Jiang and Zeng, 2015; Schmidt et al., 2015; Martín-Martínez et al., 2015; Chen et al., 2017; Lai et al., 2020; Pan et al., 2016; Chou et al., 2020) between 6 to 10 weeks; and 8 studies (Lakes et al., 2013; Crova et al., 2014; Alesi et al., 2016; Pesce et al., 2016; Cho et al., 2017; Dai, 2020; Ma et al., 2022; Yin et al., 2017) between 16 to 36 weeks.

The intervention frequency ranged from once a week (Crova et al., 2014; Pesce et al., 2016) to five times a week (Cho et al., 2017; Dai, 2020). Among them, there were 11 studies with a frequency of 1 to 2 times per week (Chang et al., 2013; Lakes et al., 2013; Crova et al., 2014; Jiang and Zeng, 2015; Schmidt et al., 2015; Martín-Martínez et al., 2015; Alesi et al., 2016; Pesce et al., 2016; Chen et al., 2017; Lai et al., 2020); and 6 studies with a frequency of 3 to 5 times per week (Cho et al., 2017; Dai, 2020; Ma et al., 2022; Pan et al., 2016; Yin et al., 2017; Chou et al., 2020). The duration of each session ranged from 30 min per session (Pan et al., 2016; Yin et al., 2017) to 120 min per session (Crova et al., 2014; Dai, 2020). Specifically, there were 2 studies with a duration of 30 min per session (Pan et al., 2016; Yin et al., 2017); 12 studies with a duration of 30 to 60 min per session (Chang et al., 2013; Lakes et al., 2013; Jiang and Zeng, 2015; Schmidt et al., 2015; Martín-Martínez et al., 2015; Pesce et al., 2016; Chen et al., 2017; Cho et al., 2017; Lai et al., 2020; Ma et al., 2022; Chou et al., 2020); and 3 studies with a duration of 75 to 120 min per session (Crova et al., 2014; Alesi et al., 2016; Dai, 2020).

The control measures included traditional physical education classes (Lakes et al., 2013; Crova et al., 2014; Schmidt et al., 2015; Alesi et al., 2016; Pesce et al., 2016; Chen et al., 2017; Pan et al., 2016; Chou et al., 2020), basic academic courses (Lai et al., 2020), aerobic exercises (Martín-Martínez et al., 2015), and a no-treatment control group (Jiang and Zeng, 2015; Cho et al., 2017; Dai, 2020; Ma et al., 2022; Yin et al., 2017).

#### 4.2.4 Outcome variables

15 studies have explored the effects of open-skill exercise interventions on inhibitory control. Among them, 10 studies used reaction time to assess task performance, including the Flanker task (Chang et al., 2013; Schmidt et al., 2015; Chen et al., 2017; Dai, 2020; Pan et al., 2016; Yin et al., 2017), Hearts-and-Flowers task (Lakes et al., 2013), GO/NOGO (Ma et al., 2022), and Stroop (Chou et al., 2020); 5 studies used accuracy to assess task performance, including the Flanker (Chang et al., 2013), Hearts-and-Flowers task (Lakes et al., 2013), Stroop (Martín-Martínez et al., 2015), and GO/NO GO (Ma et al., 2022); 4 studies used scores to assess task performance, including RNG (Crova et al., 2014; Pesce et al., 2016) and Stroop (Jiang and Zeng, 2015; Cho et al., 2017). 12 studies have investigated the effects of open-skill exercise interventions on working memory. Among them, 6 studies used reaction time to assess task performance, including 1-back (Chen et al., 2017; Lai et al., 2020; Ma et al., 2022; Pan et al., 2016; Yin et al., 2017) and 2-back (Dai, 2020; Ma et al., 2022); 2 studies used accuracy to assess task performance, including 1-back (Schmidt et al., 2015; Ma et al., 2022) and 2-back (Ma et al., 2022); 5 studies used scores to assess task performance, including RNG (Crova et al., 2014; Pesce et al., 2016), Corsi blocks test (Jiang and Zeng, 2015; Alesi et al., 2016), Digital and letter span (Martín-Martínez et al., 2015), Forward Digit Span (Alesi et al., 2016), and Backward Digit Span (Alesi et al., 2016). 9 studies have explored the effects of open-skill exercise interventions on cognitive flexibility. Among them, 8 studies used reaction time to assess task performance, including the Hearts-and-Flowers task (Lakes et al., 2013), More-odd shifting (Schmidt et al., 2015; Chen et al., 2017; Ma et al., 2022; Pan et al., 2016; Yin et al., 2017), Trail Making Test (Martín-Martínez et al., 2015), and Salthouse (Dai, 2020); 2 studies used accuracy to assess task performance, including the Hearts-and-Flowers task (Lakes et al., 2013) and More-odd shifting (Ma et al., 2022); 1 study used scores to assess task performance, including the flexible item selection task (Jiang and Zeng, 2015).

The basic information of the included studies is detailed in Table 1.

**Table 1:** Summary of original data information included in the meta-analysis.

Included studies and design	Participant characteristics (*n*/age/F%/ethnicity)	Intervention and control measures	Outcome variables	
			Tools	Results
Chang et al. (2013) QED	T = 13/7.2 ± 0.3y/46.2% Chinese Taiwan	8 weeks of low-intensity (40 ~ 50% HRmax) soccer (ST) training, 2 times/week, 35 min/session vs. pre-test	①Flanker#&	+
Chang et al. (2013) QED	T = 13/7.0 ± 0.3y/53.9% Chinese Taiwan	8 weeks of moderate-intensity (60 ~ 70% HRmax) soccer (ST) training, 2 times/week, 35 min/session vs. pre-test	①Flanker#&	+
Lakes et al. (2013) RCT	T = 50/12.2y/52.00% C = 31/12.3y/48.00% Americans	36 weeks of taekwondo (IN), 2 times/week, 45 min/session (T) vs. traditional physical education (C)	①Hearts-and-Flowers task- incongruent #& ③Hearts-and-Flowers task-mixed #&	0 0
Crova et al. (2014) RCT	T = 37/9.6 ± 0.5y/46.0% C = 33/9.6 ± 0.5y/54.6% Italians	21 weeks of moderate-intensity (150.5 ± 6.4 bpm) tennis (IN), 1 time/week, 120 min/session (T) vs. traditional physical education (C)	①RNG- Inhibition mean index ^ ②RNG- Working memory refreshing mean index ^	0 +
Jiang and Zeng (2015) RCT	T = 31/5 ~ 6y/NC C = 30/5 ~ 6y/NC Chinese	8 weeks of moderate-intensity (60 ~ 70% HRmax) soccer game (ST), 2 times/week, 35 min/session (T) vs. blank control (C)	①Stroop^ ②Corsi blocks test^ ③Flexible item selection task ^	+ 0 0
Schmidt et al. (2015) RCT	T = 69/11.3 ± 0.6y/62.3% C = 55/11.4 ± 0.6y/49.1% Swiss	6 weeks of high-intensity floor hockey and basketball games (ST), 2 times/week, 45 min/session (T) vs. traditional physical education (C)	①Flanker# ②2-back& ③More-odd shifting#	0 0 +
Martín-Martínez et al. (2015) QED	54/15 ~ 16y/25.9% Spanish	8 weeks of moderate-intensity (RPE = 13.36 ± 1.39) team ball games (ST), 2 times/week, 30 ~ 60 min/session (T) vs. aerobic exercise (C)	①Stroop& ②Digital and letter span^ ③Trail Making Test#	+ + +
Alesi et al. (2016) QED	T = 24/8.8 ± 1.1y/0.0% C = 20/9.3 ± 0.9y/0.0% Italians	24 weeks of soccer (ST) intervention, 2 times/week, 75 min/session (T) vs. traditional physical education, 1 time/week, 60 min/session (C)	②Forward Digit Span^ ②Backward Digit Span^ ②Corsi blocks test^	0 0 +
Pesce et al. (2016) RCT	T = 232/5 ~ 10y/50.4% C = 228/5 ~ 10y/49.6% Italians	24 weeks of moderate-intensity (131.9 ± 17.4 bpm) skill games focused on motor coordination and cognitive engagement (ST), 1 time/week, 60 min/session (T) vs. traditional physical education (C)	①RNG- Inhibition mean index^ ②RNG- Working memory refreshing mean index^	+ 0
Chen et al. (2017) RCT	T = 21/9.4 ± 0.5y/47.6% C = 20/9.2 ± 0.4y/50.0% Chinese	8 weeks of moderate-intensity (60 ~ 69% HRmax) soccer (ST) intervention, 2 times/week, 40 min/session (T) vs. traditional physical education (C)	①Flanker# ②1-back# ③More-odd shifting#	+ + +
Cho et al. (2017) RCT	T = 15/11.2 ± 0.8y/40.0% C = 15/11.3 ± 0.7y/40.0% Koreans	16 weeks of moderate-intensity (RPE = 11 ~ 15) taekwondo (IN) intervention, 5 times/week, 60 min/session (T) vs. blank control (C)	①Stroop^	+
Dai (2020) QED	T = 46/10.5 ± 0.3y/NC C = 43/10.4 ± 0.3y/NC Chnese	24 weeks of moderate-intensity (60 ~ 69% HRmax) soccer (ST) intervention, 5 times/week, 120 min/session (T) vs. blank control (C)	①Flanker# ②2-back# ③Salthouse#	+ + +
Lai et al. (2020) RCT	T = 10/5 ~ 7y/50.0% C = 10/5 ~ 7y/50.0% Chinese	8 weeks of moderate-intensity (60 ~ 69% HRmax) tennis (IN) intervention, 2 times/week, 60 min/session (T) vs. basic academic curriculum (C)	②1-back#	+
Ma et al. (2022) QED	T = 40/9.2 ± 0.3y/NC C = 40/9.2 ± 0.3y/NC Chinese	16 weeks of soccer (ST) intervention, 3 times/week, 40 min/session (T) vs. blank control (C)	①GO/NO GO# ①GO/NO GO& ②1-back# ②1-back& ②2-back# ②2-back& ③More-odd shifting# ③More-odd shifting&	0 + + 0 + 0 0 +
Pan et al. (2016) RCT	T = 25/12.0 ± 0.6y/NC C = 23/12.0 ± 0.6y/NC Chinese	10 weeks of moderate-intensity (60 ~ 69% HRmax) basketball (ST) intervention, 3 times/week, 30 min/session (T) vs. traditional physical education (C)	①Flanker# ②1-back# ③More-odd shifting#	+ + +
Yin et al. (2017) RCT	T = 26/10 ~ 11y/NC C = 21/10 ~ 11y/NC Chinese	16 weeks of moderate-intensity (60 ~ 69% HRmax) basketball (ST) intervention, 3 times/week, 30 min/session (T) vs. blank control (C)	①Flanker# ②1-back# ③More-odd shifting#	+ + +
Chou et al. (2020) RCT	T = 44/12.3 ± 0.7y/38.6% C = 40/12.1 ± 0.7y/37.5% Chinese Taiwan	8 weeks of moderate-intensity (around 150 bpm) playful running and jumping, baseball, soccer, and basketball (ST), 3 times/week, 40 min/session (T) vs. traditional physical education	①Stroop#	+

*n* represents the sample size; F% represents the proportion of female participants; T = Experimental Group; C = Control Group; RCT = Randomized Controlled Trial; QED = Quasi-Experimental Design; y = years; NC = Not Clear; HRmax = Maximum Heart Rate; bpm = Beats Per Minute (referring to heart rate); RPE = Rating of Perceived Exertion; #Reaction Time: This symbol is used to denote the time taken to respond to a stimulus or complete a task; &Accuracy: This symbol represents the correctness or precision of the responses or results; ^Score: This symbol indicates the numerical value or points achieved in a test or assessment; a, b = Two different sub-studies within the same study; ST = Strategic Skills; IN = Interceptive Skills; ① = Inhibitory Control; ② = Working Memory; ③ = Cognitive Flexibility; + Indicates a benefit to the experimental group; 0 Indicates no significant difference between the experimental and control groups; Pre = Pre-test; Post = Post-test; RNG = Random Number Generation Task.

### 4.3 Quality assessment results

In the 11 RCTs, 4 studies reported on randomization methods, mainly including whole group randomization (Crova et al., 2014; Pan et al., 2016; Pesce et al., 2016) and lottery method (Chen et al., 2017). Five studies reported on the implementation strategies of blinding, mainly including blinding of assessors (Lakes et al., 2013; Schmidt et al., 2015) and double blind (Chen et al., 2017; Lai et al., 2020; Chou et al., 2020). Six studies reported on the completeness of outcome data, indicating no participant dropouts or missing participants. Five studies reported participant dropouts, with the dropout sample ranging from 7 to 17 cases. Additionally, no study detailed the strategy of allocation concealment, and there was no instance of selective reporting of study results, and it is unclear whether other biases exist. The quality assessment results of the RCTs are detailed in Table 2. The quality scores of the six QEDs ranged from 17 to 19, and the quality of the included literature was relatively high (Table 3). The main reasons for the lower quality scores were that the endpoint indicator evaluation was not implemented using a blinded method, sample size was not estimated, the proportion of dropouts/missed visits was >5% and the reasons for presentation/missed visits were not stated.

**Table 2:** Results of risk of bias assessment for RCT studies.

Included studies	Randomization methods	Blinding	Allocation concealment	Integrity of result data	Selective reporting of study results	Other biases
Lakes et al. (2013)	Unclear	Blinding of assessors	Unclear	Complete	No	Unclear
Crova et al. (2014)	Whole group randomization	Unclear	Unclear	Complete	No	Unclear
Jiang and Zeng (2015)	Unclear	Unclear	Unclear	Complete	No	Unclear
Schmidt et al. (2015)	Unclear	Blinding of assessors	Unclear	8.6% missed visits	No	Unclear
Pan et al. (2016)	Whole group randomization	Unclear	Unclear	Complete	No	Unclear
Pesce et al. (2016)	Stratified whole-group randomization	Unclear	Unclear	17 cases of missed visits	No	Unclear
Chen et al. (2017)	Lottery method	Double blind	Unclear	7 cases of missed visits	No	Unclear
Yin et al. (2017)	Unclear	Unclear	Unclear	13 cases of missed visits	No	Unclear
Cho et al. (2017)	Unclear	Unclear	Unclear	Complete	No	Unclear
Lai et al. (2020)	Unclear	Double blind	Unclear	12 cases of missed visits	No	Unclear
Chou et al. (2020)	Unclear	Double blind	Unclear	Complete	No	Unclear

**Table 3:** Results of risk of bias assessment for QED studies.

Included studies	(1)	(2)	(3)	(4)	(5)	(6)	(7)	(8)	(9)	(10)	(11)	(12)	Total
Chang et al. (2013)	2	2	2	2	0	2	2	0	2	2	1	2	19
Chang et al. (2013)	2	2	2	2	0	2	2	0	2	2	1	2	19
Martín-Martínez et al. (2015)	2	1	2	2	0	2	2	0	2	2	2	2	19
Alesi et al. (2016)	2	1	2	2	0	2	2	0	2	2	2	2	19
Dai (2020)	2	1	2	2	0	2	0	0	2	2	2	2	17
Ma et al. (2022)	2	0	2	2	0	2	1	0	2	2	2	2	17

(1) the study purpose is clearly given; (2) consistency of the included subjects; (3) collection of expected data; (4) the outcome indicators can properly reflect the study purpose; (5) objectivity of outcome index evaluation; (6) whether the follow-up time is sufficient; (7) the rate of lost visits is lower than 5%; (8) whether the sample size has been estimated; (9) whether the control group has been properly selected; (10) whether the control group is synchronized; (11) whether the baseline between groups is comparable; (12) whether the statistical analysis is appropriate; NA = not applicable.

### 4.4 Main effect tests

The studies on the effects of open-skill exercise interventions on inhibitory control, working memory, and cognitive flexibility all exhibit high heterogeneity (*I*^2^ > 80%, *p* < 0.01), hence a random-effects model was used for the main effect test. The results (Table 4) show that open-skill exercise interventions have positive effects (*p* < 0.01) on inhibitory control (*SMD* = −0.627, 95%*CI* = −0.890 to −0.363), working memory (*SMD* = −0.517, 95%*CI* = −0.768 to −0.266), and cognitive flexibility (*SMD* = −0.652, 95%*CI* = −1.085 to −0.219).

**Table 4:** The effects of open-skill exercise intervention on the executive functions of children and adolescents.

Variables	*k*	Heterogeneity test		Main effect test			
		*I^2^*	*P*	*SMD*	95%*CI*	*Z*	*P*
Inhibitory control	19	82.3	0.000	−0.627	(−0.890, −0.363)	4.66	0.000
Working memory	17	80.3	0.000	−0.517	(−0.768, −0.266)	4.03	0.000
Cognitive flexibility	11	88.8	0.000	−0.652	(−1.085, −0.219)	2.95	0.003

*k represents the number of studies included in the meta-analysis for each variable.*

### 4.5 The moderating effect of open-skill types

The examination of the moderating effect of open-skill types (Table 5) shows that there is a moderating effect of open-skill types on the effect of exercise intervention in inhibitory control (*Q* = 15.86, *p* < 0.01). Specifically, strategic skill exercise intervention has a positive effect on inhibitory control (*SMD* = −0.707, 95%*CI* = −0.819 to −0.594), while the effect of interceptive skill exercise intervention is not significant (*p* > 0.05). There is no moderating effect of open-skill types on the effects of exercise intervention on working memory and cognitive flexibility (*p* > 0.05), but both strategic and interceptive skill exercise interventions have positive effects (*p* < 0.05).

**Table 5:** Examination of the moderating effect of open-skill types.

Variables	Skill types	*k*	Heterogeneity test		Main effect test			
			*Q*	*P*	*SMD*	95%*CI*	*Z*	*P*
Inhibitory control	Strategic	15	15.86	0.000	−0.707	(−0.819, −0.594)	12.35	0.000
	Interceptive	4			−0.136	(−0.394, 0.121)	1.04	0.298
Working memory	Strategic	16	0.24	0.621	−0.394	(−0.499, −0.288)	7.31	0.000
	Interceptive	1			−0.517	(−0.994, −0.040)	2.12	0.034
Cognitive flexibility	Strategic	9	1.10	0.294	−0.614	(−0.769, −0.458)	7.74	0.000
	Interceptive	2			−0.402	(−0.765, −0.038)	2.17	0.030

*k represents the number of studies included in the meta-analysis for each variable.*

### 4.6 The moderating effect of quantitative intervention elements

Firstly, the intensity level plays a moderating role in the effect of open-skill exercise interventions on inhibitory control (*Q* = 15.10, *p* < 0.01), with moderate intensity showing a positive intervention effect (*SMD* = −0.762, 95%*CI* = −0.886 to −0.638), while low and high intensities do not show significant intervention effects (*p* > 0.05). Additionally, moderate intensity has positive intervention effects (*p* < 0.01) on working memory (*SMD* = −0.504, 95%*CI* = −0.635 to −0.372) and cognitive flexibility (*SMD* = −0.750, 95%*CI* = −0.956 to −0.543), while high intensity does not significantly affect working memory (*p* > 0.05). Secondly, the period of the intervention has a moderating effect on the efficacy of open-skill exercise interventions for working memory (*Q* = 7.72, *p* < 0.01), with both 6 to 10 weeks and 16 to 36 weeks showing positive intervention effects (*p* < 0.01), but the 6 to 10 weeks period having a higher effect size (*SMD* = −0.653, 95%*CI* = −0.860 to −0.447). Furthermore, both periods significantly affect inhibitory control and cognitive flexibility (*p* < 0.01).

Thirdly, The frequency of exercise has a moderating effect on the efficacy of open-skill exercise interventions for inhibitory control (*Q* = 4.08, *p* < 0.05), with both 1 to 2 times per week and 3 to 5 times per week showing positive intervention effects (*p* < 0.01), but 3 to 5 times per week having a higher effect size (*SMD* = -0.782, 95%*CI* = -0.973 to −0.590). Both frequencies significantly affect working memory and cognitive flexibility (*p* < 0.01). Finally, the duration of each exercise session has a moderating effect on the efficacy of open-skill exercise interventions for working memory (*Q* = 24.03, *p* < 0.01) and cognitive flexibility (*Q* = 16.84, *p* < 0.01), with all durations from 30 min, 30 to 60 min, and 75 to 120 min showing positive intervention effects, but 30 min showing a higher effect size for working memory (*SMD* = −1.461, 95%*CI* = −1.918 to −1.005), and 75 to 120 min for cognitive flexibility (*SMD* = −1.474, 95%*CI* = −1.944 to −1.004); moreover, all durations significantly affect inhibitory control (*p* < 0.01). The test for the moderating effect of quantitative intervention elements is detailed in Table 6.

**Table 6:** Examination of the moderating effect of quantitative intervention elements.

Variables	Elements	Types	*k*	Heterogeneity test		Main effect test			
				*Q*	*P*	*SMD*	95%*CI*	*Z*	*P*
Inhibitory control	Intensity	Low	2	15.10	0.001	−0.541	(−1.095, 0.013)	1.91	0.056
		Moderate	12			−0.762	(−0.886, −0.638)	12.03	0.000
		High	1			−0.024	(−0.378, 0.331)	0.13	0.897
	Period	6 ~ 10 weeks	10	1.06	0.303	−0.544	(−0.714, −0.374)	6.27	0.000
		16 ~ 36 weeks	9			−0.656	(−0.785, −0.527)	9.98	0.000
	Frequency	1 ~ 2 times/week	12	4.08	0.043	−0.548	(−0.670, −0.426)	8.81	0.000
		3 ~ 5times/week	7			−0.782	(−0.973, −0.590)	8.01	0.000
	Duration	30 min/times	2	0.53	0.768	−0.762	(−1.181, −0.343)	3.57	0.000
		30 ~ 60 min/times	15			−0.609	(−0.721, −0.497)	10.64	0.000
		75 ~ 120 min/times	2			−0.581	(−0.904, −0.258)	3.53	0.000
Working memory	Intensity	Moderate	9	1.58	0.209	−0.504	(−0.635, −0.372)	7.50	0.000
		High	1			−0.260	(−0.616, 0.096)	1.43	0.152
	Period	6 ~ 10 weeks	6	7.72	0.005	−0.653	(−0.860, −0.447)	6.20	0.000
		16 ~ 36 weeks	11			−0.315	(−0.434, −0.196)	5.19	0.000
	Frequency	1 ~ 2 times/week	10	0.04	0.849	−0.393	(−0.518, −0.267)	6.12	0.000
		3 ~ 5times/week	7			−0.414	(−0.595, −0.233)	4.49	0.000
	Duration	30 min/times	2	24.03	0.000	−1.461	(−1.918, −1.005)	6.27	0.000
		30 ~ 60 min/times	10			−0.303	(−0.421, −0.184)	5.00	0.000
		75 ~ 120 min/times	5			−0.499	(−0.733, −0.264)	4.16	0.000
Cognitive flexibility	Intensity	Moderate	6	2.08	0.149	−0.750	(−0.956, −0.543)	7.11	0.000
		High	1			−0.445	(−0.803, −0.806)	2.43	0.015
	Period	6 ~ 10 weeks	5	0.36	0.649	−0.536	(−0.741, −0.331)	5.12	0.000
		16 ~ 36 weeks	6			−0.624	(−0.823, −0.424)	6.13	0.000
	Frequency	1 ~ 2 times/week	6	3.74	0.053	−0.462	(−0.649, −0.275)	4.84	0.000
		3 ~ 5times/week	5			−0.748	(−0.969, −0.527)	6.62	0.000
	Duration	30 min/times	2	16.84	0.000	−0.738	(−1.157, −0.320)	3.46	0.001
		30 ~ 60 min/times	8			−0.453	(−0.614, −0.293)	5.53	0.000
		75 ~ 120 min/times	1			−1.474	(−1.944, −1.004)	6.15	0.000

*k represents the number of studies included in the meta-analysis for each variable.*

### 4.7 The moderating effect of demographic variables

Firstly, age has a moderating effect on the efficacy of open-skill exercise interventions on inhibitory control and working memory (*p* < 0.01). Although open-skill exercises have positive intervention effects on both inhibitory control and working memory for the 5 to 9 and 10 to 16 age groups (*p* < 0.01), the promotion effect on inhibitory control is more pronounced for the 5 to 9 age group (*SMD* = −0.764, 95%*CI* = −0.901 to −0.627), and the promotion effect on working memory is more pronounced for the 10 to 16 age group (*SMD* = −0.758, 95%*CI* = −0.960 to −0.555). Additionally, open-skill exercises have positive intervention effects on cognitive flexibility for both the 5 to 9 and 10 to 16 age groups (*p* < 0.01). Secondly, ethnicity has a moderating effect on the efficacy of open-skill exercise interventions on inhibitory control (*p* < 0.01). Although open-skill exercises have positive intervention effects on inhibitory control for both Eastern and Western groups (*p* < 0.01), the promotion effect on inhibitory control is more pronounced for the Eastern group (*SMD* = −0.831, 95%*CI* = −0.991 to −0.671). Furthermore, open-skill exercises have positive intervention effects on working memory and cognitive flexibility for both Eastern and Western groups (*p* < 0.01). The moderating effects of age and ethnicity are detailed in Table 7.

**Table 7:** Examination of the moderating effect of age and ethnicity.

Variables	Elements	Types	*k*	Heterogeneity test		Main effect test			
				*Q*	*P*	*SMD*	95%*CI*	*Z*	*P*
Inhibitory control	Age	5 ~ 9 years	10	10.40	0.001	−0.764	(−0.901, −0.627)	10.95	0.000
		10 ~ 16 years	9			−0.423	(−0.579, −0.267)	5.32	0.000
	Ethnicity	Eastern	13	11.83	0.001	−0.831	(−0.991, −0.671)	10.18	0.000
		Western	6			−0.464	(−0.598, −0.330)	6.78	0.000
Working memory	Age	5 ~ 9 years	12	16.28	0.000	−0.274	(−0.394, −0.154)	4.47	0.000
		10 ~ 16 years	5			−0.758	(−0.960, −0.555)	7.34	0.000
	Ethnicity	Eastern	10	0.07	0.786	−0.417	(−0.580, −0.254)	5.02	0.000
		Western	7			−0.388	(−0.521, −0.255)	5.70	0.000
Cognitive flexibility	Age	5 ~ 9 years	4	1.53	0.216	−0.455	(−0.700, −0.209)	3.63	0.000
		10 ~ 16 years	7			−0.646	(−0.821, −0.470)	7.20	0.000
	Ethnicity	Eastern	7	2.57	0.109	−0.687	(−0.880, −0.494)	6.98	0.000
		Western	4			−0.452	(−0.665, −0.240)	4.17	0.000

*k represents the number of studies included in the meta-analysis for each variable.*

A meta-regression analysis was conducted to explore the moderating effect of the continuous variable gender ratio, and the results (Table 8) show that gender ratio does not have a moderating effect on the effects of open exercise intervention on inhibitory control, working memory, and cognitive flexibility (*p* > 0.05).

**Table 8:** Examination of the moderating effect of gender ratio.

Variables	*β*	95*%CI*	*t*	*P*
Inhibitory control	0.996	(0.945, 1.048)	−0.17	0.870
Working memory	1.001	(0.980, 1.022)	0.09	0.932
Cognitive flexibility	0.999	(0.857, 1.166)	−0.01	0.989

### 4.8 Publication bias test

A linear regression equation was constructed with the effect size as the dependent variable and the precision of the effect estimate as the independent variable. The intercept of the regression equation represents the bias. The closer it is to 0, the less likely there is publication bias. If *p* > 0.05 and the 95%*CI* includes 0, it indicates no publication bias (Sun and Shi, 2021). The results (Table 9) show that inhibition control, working memory, and cognitive flexibility have *p* > 0.05 and the 95%*CI* includes 0, indicating that there is no publication bias in the included studies, and the meta-analysis results are stable and reliable.

**Table 9:** Results of egger’s linear regression test for publication bias.

Variables	*β*	*SE*	*t*	*P*	95%*CI*
Inhibitory control	0.004	1.406	0.00	0.998	(−2.962, 2.970)
Working memory	−2.056	1.323	−1.55	0.141	(−4.877, 0.765)
Cognitive flexibility	−5.091	5.121	−0.99	0.346	(−16.675, 6.494)

### 4.9 Sensitivity analysis

After sequentially excluding each study, the results of the combined effect test did not undergo any fundamental changes, thus indicating no issue with sensitivity.

## 5 Discussion

### 5.1 Open-skill exercise has positive benefits on executive function

Open-skill exercise has a positive intervention effect on inhibitory control, working memory, and cognitive flexibility of children and adolescents, supporting the research hypothesis 1. The results of this study are similar to those of Feng et al. (2023) and Shi et al. (2022), which all support the positive effects of open-skill exercise. The positive benefits of open-skill exercise can be explained as follows. Firstly, open-skill exercises typically involve constantly changing environmental stimuli, requiring participants to maintain a high level of alertness and adaptability to make flexible decisions in different scenarios. The complexity and unpredictability of such environments compel the brain to continuously process new information, thereby enhancing executive functions. Secondly, open-skill exercises often require the execution of complex and variable movement sequences, demanding that participants have a high capacity for working memory to remember, manipulate, and update information to guide actions. The continuous demand and challenge to working memory help to improve its efficiency and capacity (Shi et al., 2019; Teitelbaum and Teitelbaum, 2007). Relevant studies (Mavilidi et al., 2015; Moreau et al., 2015) have shown that this kind of complex exercise can increase the number of Purkinje neurons and synapses, promote angiogenesis in the prefrontal cortex, improve neural function remodeling, and activate the visual–spatial network and the sensorimotor network related to the executive control system. Finally, long-term regular open-skill exercise can enhance an individual’s cardiorespiratory fitness, thereby increasing the capillary density of brain tissue (Voelcker-Rehage et al., 2011), which further promotes the development of executive function in children and adolescents.

### 5.2 Differences in the executive function of strategic and interceptive skills exercise interventions

The results of this study indicate that the effect of strategic skill exercise intervention on the executive function of children and adolescents is better than that of interceptive skills, especially in the dimension of inhibitory control, supporting the research hypothesis 2. The environment of strategic skill exercises such as soccer and basketball is rapidly changing, with quick spatial and temporal transitions and intense physical confrontation, requiring individuals to process information from the identities of players on the field, their movement characteristics, and the trajectory of the ball within a limited time (Shi et al., 2023b). Compared to interceptive skills, the environment is richer in environmental stimuli and interpersonal interaction information. Therefore, the unpredictability of the strategic skill exercise environment is higher, necessitating more conscious and cognitive participation from the subjects, posing a stronger cognitive challenge to children and adolescents. Specifically, during the exercise, the participants need to use the selective attention control system to filter out irrelevant information, maintain attention to key information, and continuously refresh and store scene information through the working memory system to flexibly make sports decisions, technical choices, and movement changes (Shi et al., 2023a). Research on the relationship between executive function and sports decision-making has also confirmed this, that more indicators of executive function dimensions are associated with tactical decision-making in basketball (strategic skills) (Lu, 2021), while only inhibitory control is associated with sports decision-making in badminton (interceptive skills) (Dong, 2015).

Relevant empirical studies have also confirmed the main viewpoints of this study. Krenn et al. (2018) compared the executive function performance of athletes with strategic, interceptive, and closed skills and found that, compared to athletes with closed skills, those with strategic skills showed unique cognitive advantages in inhibitory control, working memory, and cognitive flexibility; while athletes with interceptive skills only performed well in inhibitory control functions. Yu et al. (2019) used the Attention Network Test and near-infrared brain imaging technology to examine the behavioral and brain activation differences in attentional executive control network functions among basketball (strategic skills), table tennis (interceptive skills), and track and field (closed skills) athletes. They found that basketball and table tennis athletes showed higher executive control advantages, accompanied by significant activation in the right prefrontal cortex and the inferior frontal gyrus; moreover, compared to table tennis athletes, basketball athletes showed more brain area activation in the frontal lobe association areas. Wu et al. (2015) compared the brain gray matter differences between badminton (interceptive skills) and basketball (strategic skills) athletes and found that the brain structure of athletes has project-specific attributes. Badminton athletes had significantly increased gray matter volume in the left inferior frontal gyrus, left superior parietal lobule, and left precuneus, which are related to fine motor control and perceptual spatial positioning (Baumgartner et al., 2013; Macuga and Frey, 2014); whereas basketball athletes had significantly increased gray matter volume in the middle temporal gyrus, left middle frontal gyrus, left inferior frontal gyrus, midcingulate gyrus, and insula, which are related to visual information processing, response inhibitory control, and perceptual-motor decision-making (Creem and Proffitt, 2001; Simmons et al., 2012). Therefore, the activation of more regions by strategic skill exercises is related to the improvement of executive function behavioral performance.

### 5.3 Quantitative intervention elements have a moderating effect on the effects of open-skill exercise

Firstly, the effect of moderate-intensity exercise intervention on the executive functions of children and adolescents is optimal, which is consistent with previous studies (Chen et al., 2014; Feng et al., 2023) and supports the research hypothesis 3. According to the inverted U-shaped hypothesis, moderate-intensity exercise provides a moderate cognitive load, which can stimulate the brain, promote the production of brain-derived neurotrophic factor (BDNF) in certain brain regions, improve the supply of oxygen and nutrients to the brain, and avoid excessive intensity that could lead to cognitive overload and fatigue, which would be detrimental to the accumulation of executive function benefits (Bodensohn et al., 2024; Ferreira et al., 2011; Pereira et al., 2021). In addition, the brain during moderate-intensity exercise can allocate more resources to cognitive tasks such as attention, while high-intensity exercise consumes more cognitive resources in limb movements and is unable to allocate more cognitive resources to the processing of external information (Hagger et al., 2010; Harris and Bray, 2019). Especially for open skills with rich external environmental information, it is not conducive to the improvement of sports decision-making and the acquisition of executive functions.

Secondly, both shorter (6 ~ 10 weeks) and longer (16 ~ 36 weeks) interventions have positive effects on all dimensions of executive function, but the shorter intervention has a more positive effect on working memory, which deviates from research hypothesis 4. Liu et al. (2020) also found that exercise interventions within 12 weeks and beyond 12 weeks are both conducive to the improvement of executive function, which is similar to some of the results of this study. In addition, at the beginning of the intervention, participants may feel novelty and excitement about the new exercise program, which can increase their participation and motivation. The brain quickly adapts to the exercise, thereby improving the performance of working memory tasks in the short term. However, in the longer intervention period, participants may become accustomed to the exercise program, reducing the novelty and challenge of the exercise, and even experiencing fatigue, stress, or a decrease in motivation, which may reduce the positive impact on working memory.

Thirdly, both lower (1 ~ 2 times per week) and higher (3 ~ 5 times per week) exercise frequencies have positive intervention effects on all dimensions of executive functions, with the higher frequency showing superior benefits, which supports research hypothesis 5. Furthermore, the results of this study are consistent with previous systematic reviews and meta-analyses (Liu et al., 2020; Shi et al., 2022; Xue et al., 2019), all of which suggest that more frequent exercise interventions can lead to greater benefits in executive functions for children and adolescents. Engaging in higher frequency exercise for children and adolescents means that their brain activation and neural network remodeling occur more frequently, and this cumulative effect may promote further enhancement of executive functions (Won et al., 2021). Additionally, more frequent exercise can help children and adolescents develop stable exercise habits, which are associated with better self-control and planning, the core components of the executive control system (Galla and Duckworth, 2015).

Finally, exercise durations of short (30 min per session), moderate (30 to 60 min per session), and long (75 to 120 min per session) all have positive effects on various dimensions of executive function, with 30 min per session being the most effective intervention for working memory, and 75 to 120 min per session being the most effective for cognitive flexibility. These results do not fully support the research hypothesis 6 of the study. The findings of this study also support some of the results of Liu et al. (2020), which suggest that exercise durations of less than 30 min per session are more effective for the intervention of working memory. Shorter exercise durations can provide sufficient stimulation to activate the brain without causing excessive fatigue, thus maintaining alertness and attention (Liu et al., 2020). However, Shi et al. (2022) found that moderate exercise durations overall have a more positive promotional effect, which is inconsistent with the results of this study. This study even found that longer exercise durations are better for promoting cognitive flexibility, which contradicts the fatigue hypothesis. Cognitive flexibility is a higher cognitive structure of executive function, a more complex thought process compared to inhibitory control and working memory (Logue and Gould, 2014), and therefore may require longer exercise durations to activate this deep-level cognition. In addition, the actual benefits of executive function obtained by children and adolescents are affected by a variety of regulatory variables, so the optimal exercise duration needs to be considered comprehensively. But in any case, any duration of exercise is beneficial to any dimension of executive function.

### 5.4 Age and ethnicity have a moderating effect on the benefits of open-skill exercise

Firstly, open-skill exercises have a positive effect on all dimensions of executive functions in both the 5–9 year old and 10–16 year old groups, which is consistent with the research findings of Verburgh et al. (2014). Additionally, open-skill exercises have a more positive effect on inhibitory control in the 5–9 year old group and on working memory in the 10–16 year old group, which partially supports the research hypothesis 7, but does not fully support it. Previous systematic reviews and meta-analyses have also shown inconsistent results regarding the moderating effect of age. For example, Shi et al. (2022) found that exercise in real-word settings has a better intervention effect on working memory in school-age children than in preschool children, while Liu et al. (2020) found that exercise intervention has a positive intervention effect on inhibitory control in older ages and on working memory in younger children. The latter included both acute and long-term exercise interventions, as well as a series of laboratory-based studies, not solely examining the effects of long-term open-ended motor skill exercises, hence the results are inconsistent or even opposite to those of this study. Children aged 5–9 are in a stage of rapid development, with their brains and nervous systems maturing quickly. At this stage, open-skill exercises promote the development of inhibitory control by inhibiting instinctive reactions and by trying new movements or providing new methods for problem-solving. Adolescents aged 10–16 already possess a certain level of inhibitory control, with relatively mature lower-level executive control systems, so exercise interventions at this stage may focus more on improving their working memory. However, regardless of the age group, the effects of open-skill exercises are generally beneficial.

Secondly, open-skill exercises have a positive effect on all dimensions of executive functions in both Eastern and Western groups, and especially have a more positive intervention effect on the inhibitory control of the Eastern group. This result emphasizes the differential impact of open-skill exercise interventions on children and adolescents across cultural backgrounds. Eastern cultures typically emphasize collectivism, harmony, and adherence to social norms, which may lead to the Eastern group practicing inhibitory control more frequently in daily life, such as suppressing personal desires to conform to social standards. In addition, Eastern education systems often place greater emphasis on discipline and restraint, and in cognitive patterns, they tend to cultivate introspection and self-regulation in children and adolescents. Relevant studies (Tran et al., 2015; Yang et al., 2011) have also found that the higher inhibitory control of Eastern children and adolescents benefits from their continuous self-regulation practice. Therefore, when Eastern children and adolescents engage in open-skill exercises, this cultural inclination may enhance the positive effects of the exercise on inhibitory control.

Finally, this study did not find a moderating effect of gender and does not support research hypothesis 8. Among the included studies, only one study (Alesi et al., 2016) included only male samples for discussion, and there are no original studies that explore female samples. Therefore, this study used the continuous variable of gender ratio to examine its potential moderating effect, which may have caused some interference with the results. In fact, some studies (Duff and Hampson, 2001; Hussain, 2016) have confirmed the differences in executive functions between genders, but the results are not consistent. For example, Hussain (2016) found that the executive functions of middle school boys are higher than those of girls; while Duff and Hampson (2001) found that women’s working memory is significantly better than that of men. Therefore, this inconsistency still needs to be further examined by subsequent studies.

### 5.5 Implications for future exercise practice

The results of this study can facilitate the translation of evidence into practice by implementing effective exercise interventions in school and community sports environments to promote the development of executive functions in children and adolescents. Specifically, this study provides several insights for future exercise intervention practices. Firstly, the study results show that open-skill exercise has a positive impact on the executive functions of children and adolescents, especially strategic skills. Therefore, future exercise intervention practices should combine the interests of children and adolescents and actively recommend such exercises to promote the development of executive functions. Secondly, based on the quantitative elements of exercise, appropriate exercise intensity should be chosen, and exercise frequency and duration should be reasonably adjusted according to the dimensions of executive function development. Lastly, future intervention practices should include long-term tracking and evaluation of intervention effects to determine the lasting impact of interventions and adjust intervention strategies based on assessment results. Through these insights, future exercise intervention practices can be designed and implemented more scientifically to maximize the enhancement of executive functions in children and adolescents.

### 5.6 The limitations of this study

This study is the first to quantitatively examine the effects of open skill exercise interventions on the executive functions of children and adolescents, and further explores the heterogeneity of strategic and interceptive skill intervention effects, as well as potential moderating factors, which has guiding significance for the selection of subsequent intervention measures for executive functions of children and adolescents. However, there are some limitations in this study. Firstly, the search process was limited to Chinese and English literature, and gray literature was not further included, which may lead to publication bias, but the sensitivity analysis using the one-by-one exclusion method showed that the results are robust and reliable. Secondly, the included literature has a bias risk in methodological quality, which may interfere with the results of the intervention. Thirdly, due to the limitations of the original data, there is a lack of research on interceptive skill exercise interventions for working memory and cognitive flexibility, and the conclusions still need to be further tested by subsequent studies. Finally, given the relatively small number of included studies, the generalizability of the research may be greatly compromised. Therefore, it is recommended to further accumulate primary research in this field, ensuring that the study samples are representative and cover children and adolescents of different ages, genders, and ethnic backgrounds. By continuously iterating through research evidence, the generalizability of the research results can be gradually enhanced, improving the applicability of the research conclusions.

## 6 Conclusion

This study quantitatively explored the intervention effects of open-skill exercise on the executive functions of children and adolescents, and investigated the heterogeneity of the intervention effects of strategic and interceptive skill exercises, as well as related moderating factors. The quantitative analysis results showed that open-skill exercise has a positive intervention effect on the executive functions of children and adolescents, with the intervention effect of strategic skill exercise being superior to that of interceptive skill exercise, especially in terms of inhibitory control. Moderate-intensity and higher-frequency exercises overall have a more positive effect on promoting executive functions; interventions of 6 to 10 weeks are more effective for working memory, while 30-min sessions are the most effective for working memory, and sessions lasting 75 to 120 min are the most effective for cognitive flexibility. In addition, open-skill exercise has a more positive impact on inhibitory control in the 5–9 age group and on working memory in the 10–16 age group; open-skill exercise, especially, has a more positive intervention effect on inhibitory control in the Eastern group. The above results provide a basis for the formulation of subsequent intervention measures, which helps to effectively transform the evidence into practice in school and community sports environments.

## Data Availability

The raw data supporting the conclusions of this article will be made available by the authors, without undue reservation.
